# Precision Oncology Approach for Urachal Carcinoma: A Clinical Case Report

**DOI:** 10.3390/ijms252413315

**Published:** 2024-12-12

**Authors:** Dániel Juhász, Anita Csizmarik, János Szalontai, Attila Keszthelyi, Bálint Dér, András Kubik, Miklós Szűcs, István Kenessey, Iris E. Ertl, Walter Berger, Bernhard Englinger, Shahrokh F. Shariat, Péter Nyirády, Tibor Szarvas

**Affiliations:** 1 Department of Urology, Semmelweis University, Üllői Street 78/b, H-1082 Budapest, Hungary; ju88da@yahoo.com (D.J.); csizmarik.anita@gmail.com (A.C.); szajanos@outlook.com (J.S.); attilakeszthelyi@hotmail.com (A.K.); balintder@gmail.com (B.D.); drkubikandras@gmail.com (A.K.); szucsmdr@gmail.com (M.S.); nyiradyp@gmail.com (P.N.); 2 Department of Pathology, Forensic and Insurance Medicine, Semmelweis University, H-1082 Budapest, Hungary; steveken12@yahoo.com; 3 National Cancer Registry and Centre for Biostatistics, National Institute of Oncology, H-1082 Budapest, Hungary; 4 Department of Urology, Medical University of Vienna, Comprehensive Cancer Center, A-1090 Vienna, Austria; iris.ertl@meduniwien.ac.at (I.E.E.); bernhard.englinger@meduniwien.ac.at (B.E.); sfshariat@gmail.com (S.F.S.); 5 Center for Cancer Research, Medical University of Vienna, Comprehensive Cancer Center, A-1090 Vienna, Austria; walter.berger@meduniwien.ac.at; 6 Department of Urology, Weill Cornell Medical College, New York, NY 10065, USA; 7 Department of Urology, University of Texas Southwestern, Dallas, TX 75390, USA; 8 Department of Urology, Second Faculty of Medicine, Charles University, 150 06 Prague, Czech Republic; 9 Karl Landsteiner Institute of Urology and Andrology, A-1010 Vienna, Austria; 10 Research Center for Evidence Medicine, Urology Department Tabriz University of Medical Sciences, Tabriz 5166/15731, Iran; 11 Division of Urology, Department of Special Surgery, Jordan University Hospital, University of Jordan, Amman 11942, Jordan; 12 Department of Urology, University of Duisburg-Essen and German Cancer Consortium (DKTK), Hufelandstr. 55, D-45147 Essen, Germany

**Keywords:** Urachal carcinoma, chemotherapy, immune checkpoint inhibitor (ICI) therapy, targeted therapy, DNA sequencing, drug screening, carcinoembryonic antigen (CEA)

## Abstract

Urachal cancer (UrC) is a rare disease which is mostly diagnosed late due to symptoms caused by its local invasion to the urinary bladder. Given the lack of clinical trials and guideline recommendations for systemic treatment, a molecularly informed precision oncology approach is a viable option for UrC already in the early lines of systemic treatment. While single case experiences may provide valuable reference for later decision-making, well-documented clinical experience with off-label targeted treatments is limited to a few patients. Here, we report a case of a 31-year-old female UrC patient who underwent intensive therapy with three surgeries and five lines of systemic treatments, including chemo-, checkpoint inhibitor and tyrosine kinase inhibitor therapies. In addition, next-generation sequencing (NGS) analysis and an ex vivo drug-screening analysis were performed on patient-derived tumor cells and the results were implemented into the therapeutic decision-making. Finally, serum carcinoembryonic antigen (CEA) levels proved to be helpful for therapy monitoring during the whole follow-up period.

## 1. Introduction

The urachus is the embryological remnant of the allantois, a channel between the bladder dome and the umbilicus. Urachal cancer (UrC) is a rare disease with an estimated incidence of 4/10 million inhabitants per year [1,2,3,4]. In localized stages, surgical treatments may be effective. However, in most cases, the malignancy is either diagnosed at locally advanced stages or will show rapid progression to metastatic stages requiring systemic treatment [5]. Due to the lack of clinical studies, there are no standard systemic treatments or clear guideline recommendations available. Consequently, clinical decisions are made on an individual basis and are heterogeneous [5]. Most of our current knowledge about the treatment of UrC comes from single case reports or small institutional case series. Therefore, in rare cancers, presenting individual case histories remains a valuable source of knowledge.

## 2. Case Report

### 2.1. Diagnosis

**Figure 1:**
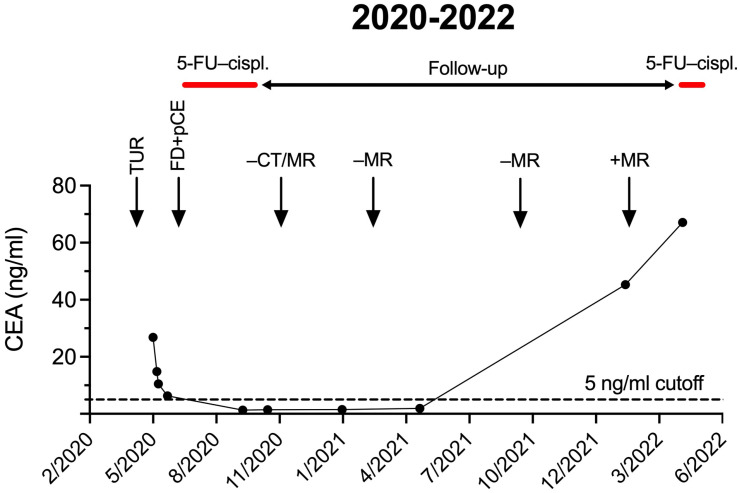
Timeline of serum CEA levels with surgical and systemic treatments and imaging follow-up examinations. Abbreviations: FD—first diagnosis, pCE—partial cystectomy.

The examination of the 32-year-old female patient began in March 2020 due to macroscopic haematuria. CT scan showed a 35 × 30 mm large lesion suspicious for UrC (Supplementary Figure S1C,D). Cystoscopy identified an area with atypical morphology at the bladder dome. Transurethral tumor resection from this location revealed intestinal adenocarcinoma. Gastrointestinal tumor invasion of the bladder was excluded by negative gastro- and colonoscopy. The initial serum carcinoembryonic antigen (CEA) tumor marker level was elevated (26.8 ng/mL, Figure 1).

### 2.2. Surgery with Curative Intent

Organ-sparing partial cystectomy (bladder dome resection) with the removal of the urachal remnant, umbilicus, and lymph node dissection was performed (Supplementary Figure S1A,B). Histological evaluation found tumor infiltration of all layers of the bladder wall without any histological evidence of lymph node metastasis. Tumor cells were found in the periurachal adipose tissue, which raised suspicion about a possible residual tumor. The postoperative CEA was 6.2 ng/mL (Figure 1) showing a reduction from the initial value. Next-generation sequencing (NGS) with the TSO500 panel (Illumina Inc., San Diego, CA, USA) identified pathogenic *TP53* (VAF: 24%), and likely pathogenic *GRM3* (VAF: 17%) and *LRP1B* (VAF: 46%) alterations along with low tumor mutational burden (TMB) and no microsatellite instability.

### 2.3. Adjuvant Systemic Therapy

To reduce the risk of recurrence due to the residual tumor, six cycles of adjuvant 5-fluorouracil (5-FU)-cisplatin chemotherapy were administered in the adjuvant setting. Between October 2020 and September 2021, multiple US, CT, and MRI imaging follow-ups were performed with negative results and serum CEA value decreased to 1.4 ng/mL, also suggesting a good therapy efficacy. For the next 10 months, multiple CT and MRI scans were negative, with CEA levels remaining low (Figure 1).

### 2.4. Recurrence and Chemotherapy Rechallenge

**Figure 2:**
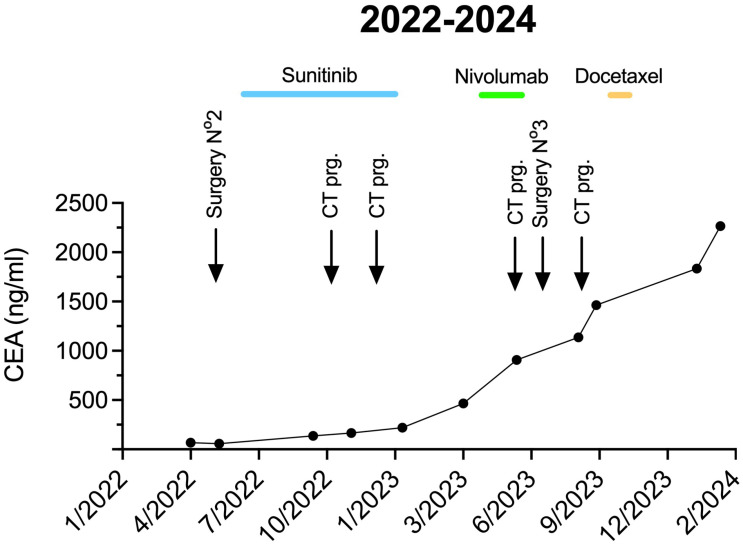
Timeline of serum CEA levels with surgical and systemic treatments and with follow-up imaging control examinations. prg. stands for progression.

In February 2022, MR examination raised the possibility of local recurrences of 12, 16, and 17 mm in size with a simultaneous increase in CEA level to 45.3 ng/mL (Figure 2). As the initial treatment with 5-FU cisplatin chemotherapy proved to be effective, we applied a rechallenge of this treatment. Following two cycles of 5-FU-cisplatin chemotherapy, further size progression of the local recurrence occurred.

### 2.5. Recurrence Surgery

In May 2022, local recurrences were removed by open abdominal surgery that discovered an unresectable minor pelvic lymph node conglomeration. Histological examination reported a mucinous adenocarcinoma with surgical margin positivity. NGS analysis of tumor tissue with the TSO500 panel identified *TP53* (VAF: 17%) mutation again, as well as *EGFR*, *RET*, and *MET* amplifications.

### 2.6. Targeted Treatments

To provide targeted therapy based on gene amplifications, we started treatment with sunitinib, a multitargeted tyrosine kinase inhibitor, for seven months until January 2023, when progression was detected by CT scan and the CEA level showed a remarkable elevation (220 ng/mL). Based on previous publications, the PD-1 inhibitor nivolumab showed efficiency in UrC [6]. Thus, we applied five cycles of nivolumab. Notably, PD-L1 immunohistochemistry found no/low PD-L1 expression (TPS = 0, CPS = 1, IC = 0) in the primary tumor tissue. In May 2023, a cystic recurrence measuring 21 cm was confirmed with a serum CEA value of 907 ng/mL (Figure 2).

### 2.7. Palliative Surgery and Further Palliative Treatments

In June 2023, a large tumor mass of 2 kg was removed from the abdomen by palliative surgery: invasion to the ovaries and small bowel was discovered intraoperatively. Viable tumor tissue from pathological residual material was cultured short-term ex vivo for drug screening purposes in order to identify potential pharmacological vulnerabilities (Supplementary Material and Methods) [7]. Multi-dose screening with 154 clinically approved anticancer compounds was performed at the Center for Cancer Research, Medical University of Vienna (Supplementary Table S1 and Supplementary Figure S2). Of note, cancer cells showed marked in vitro resistance towards those compounds previously administered to the patient, i.e., 5-FU, cisplatin, and sunitinib. Of the 154 screened drugs, 13 compounds across multiple modes of action were determined as hits, as defined by reducing cell viability of the primary tumor cell culture by at least 50% across all assay concentrations. Among those active compounds, docetaxel was the most potent with an IC_50_ of 19 nM. This finding is in agreement with a previous approach demonstrating potent antineoplastic effects of docetaxel in a patient-derived UrC cell line [8]. After losing contact with the patient until October 2023, docetaxel treatment was started but had to be interrupted after two cycles because of side effects. The patient refused further treatment, and only palliative care was provided. Due to a subsequent ileus, an acute surgical exploration was performed. Unfortunately, the extensive peritoneal carcinomatosis made colostomy creation impossible. She underwent best supportive care for a couple of weeks in our department and passed away in mid-February 2024.

## 3. Discussion

Our case represents a typical UrC history with a young patient who initially responded well to surgical therapy and adjuvant 5-FU-cisplatin chemotherapy. This is in accordance with the published literature showing that 5-FU-cisplatin combination therapies are effective in UrC [5,9]. Serum CEA level proved to be a valuable tool for therapy monitoring for the complete follow-up period and, therefore, its utilization is highly recommended. Despite multiple surgical and systemic therapies, including cytostatic chemotherapies as well as molecularly selected targeted approaches (tyrosine kinase and immune checkpoint inhibitor), a survival of 43 months could be achieved that is comparable with internationally reported median survival times [1,2,3]. This case demonstrates that UrC is an aggressive disease that could be effectively controlled for 10 months with surgical treatment and adjuvant chemotherapy, followed by a rapid progression period, which could not be effectively controlled despite aggressive treatment with three surgical and five systemic therapy lines. During the rapid progression phase of the tumor growth, in lack of treatment recommendations, therapy selection is challenging. The availability of NGS and rational therapy selection raised hopes, yet, unfortunately, DNA sequencing with even a large panel identified no well-targetable alterations. In studies utilizing NGS in retrospective UrC tissues, we found potentially targetable alterations in a high number of cases [10,11]. Therefore, we propose that clinical sequencing-based individual treatment selection represents a promising and rapidly evolving therapeutic strategy that provides potential treatment options in those (e.g., rare) tumor cases, where no systemic therapy recommendations are available. In light of the global scarcity of preclinical models of UrC, a further, novel approach is the ex vivo drug screening of primary tumor cell material for therapy selection [8]. After careful discussion with the patient and considering the exhausted treatment opportunities, we used this approach. To our knowledge, this is the first case of UrC where patient-derived ex vivo drug-screening was implemented into therapeutic decision-making. However, our case is limited in terms of representing the efficacy of this approach, as the selected drug could only be applied for a short term due to side effects, which led to the refusal of therapy by the patient.

## 4. Conclusions

Overall, UrC is an aggressive disease with limited therapy options. Molecularly- (NGS) or ex vivo screening-informed individual treatment decisions in combination with blood-based monitoring markers may improve a patient’s prognosis. However, further research and well-documented case collections are needed to provide information for improved therapeutic decision-making.

## Data Availability

The datasets used and/or analyzed during the current study are available from the corresponding author on reasonable request.

## Supplementary Materials

The following supporting information can be downloaded at: https://www.mdpi.com/article/10.3390/ijms252413315/s1.

## Abbreviations

5-FU	5-fluorouracil chemotherapy
CEA	carcinoembryonic antigen
CPS	combined positivity score (PD-L1 immunohistochemistry evaluation method)
CT	computer tomography
FDA	Food and Drug Administration
IC	immune cells (PD-L1 immunohistochemistry evaluation method)
ICI	Immune checkpoint cnhibitor
MRI	magnetic resonance imaging
NGS	next-generation sequencing
PD-L1	Programmed Death-Ligand 1
TMB	Tumor Mutational Burden
TPS	Tumor Proportion Score (PD-L1 immunohistochemistry evaluation method)
UrC	urachal cancer
US	ultrasound
VAF	Variant allele frequency
