# Supplementary material for: Precision Oncology Approach for Urachal Carcinoma: A Clinical Case Report
Source: Int J Mol Sci. 2024 Dec 12;25(24):13315. doi: 10.3390/ijms252413315 (PMC11678524; doi:10.3390/ijms252413315)
Supplement: Supplementary file 1 [file ijms-25-13315-s001.zip › Supplementary Legends.pdf]

Legends

Supplementary Figure S1.

Macroscopic pathological images (A-B), and diagnostic CT scan images of the urachal cancer (C-D). Arrows indicate the tumor at the bladder dome.

Supplementary Figure S2.

Viability curves of selected drugs with potential therapeutic effects.

Supplementary Table S1.

Complete list of compounds (n = 154) included in the drug screen, with their mode of action, molecular targets and IC50 values.
