# Supplementary material for: Precision Oncology Approach for Urachal Carcinoma: A Clinical Case Report
Source: Int J Mol Sci. 2024 Dec 12;25(24):13315. doi: 10.3390/ijms252413315 (PMC11678524; doi:10.3390/ijms252413315)
Supplement: Supplementary file 1 [file ijms-25-13315-s001.zip › Supplementary Material and Methods.pdf]

Primary Cell Culturing

For the establishment of **patient-derived tumour cells**, macroscopically visible parts of the tumour that were not required for diagnosis were obtained in the course of abdominal surgery performed at the Department of Urology of the Semmelweis University in Budapest, Hungary. Informed consent was obtained before sample collection (45764-2/2017/EKU). Subsequently, the tumour samples were sent to the Department of Urology of the Vienna General Hospital, Vienna, Austria. To ensure the viability of tumour cells, the specimens were transferred in RPMI medium on dry ice. Upon arrival, the samples were cut in small pieces and transferred to T25 culture flasks with ACL medium as previously described [1]. After seven days, cells were transferred to standard RPMI 1640 medium supplemented with 10% fetal calf serum and 1% penicillin-streptomycin (all from Thermo Fisher Scientific, Waltham, MA, USA), and expanded. For **high-throughput drug screening**, compounds were transferred into 384-well drug plates by acoustic droplet ejection using an Echo 520 liquid handler (LABCYTE, Sunnyvale, CA, USA). Cells were seeded onto drug plates at a density of $1x10^5$ cells/well. Drug screening was performed on a Cell Explorer platform (PerkinElmer, Waltham, Massachusetts, USA) using 154 compounds in 6-point dose-response curves (Suppl. Table 1), measured in duplicates. All components were diluted in 0.1% DMSO. Cell viability was measured after 72h using CellTiter-Glo® (Promega, Fitchburg, Madison, USA). The drug screen was conduced at the Molecular Discovery Platform of the CeMM Research Center for Molecular Medicine of the Austrian Academy of Sciences, Vienna, Austria.

1. Langdon SP. Formulations of Commonly Used Cell Culture Media Cancer cell culture : methods and protocols. Humana Press; 2004. 360 p
