# Supplementary figures and images for: Precision Oncology Approach for Urachal Carcinoma: A Clinical Case Report
Source: Int J Mol Sci. 2024 Dec 12;25(24):13315. doi: 10.3390/ijms252413315 (PMC11678524; doi:10.3390/ijms252413315)

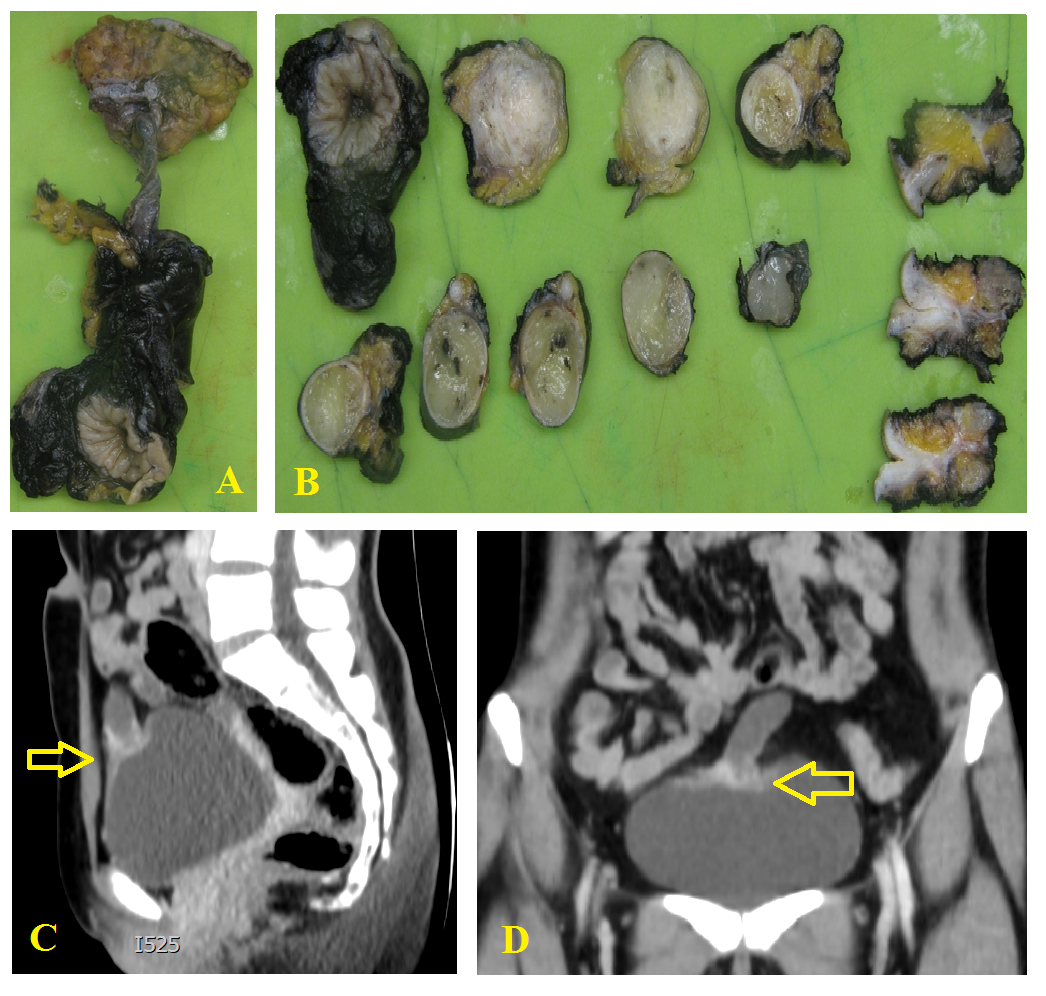

Supplement: Supplementary file 1 [file ijms-25-13315-s001.zip › Supplementary Figure S1..tif]

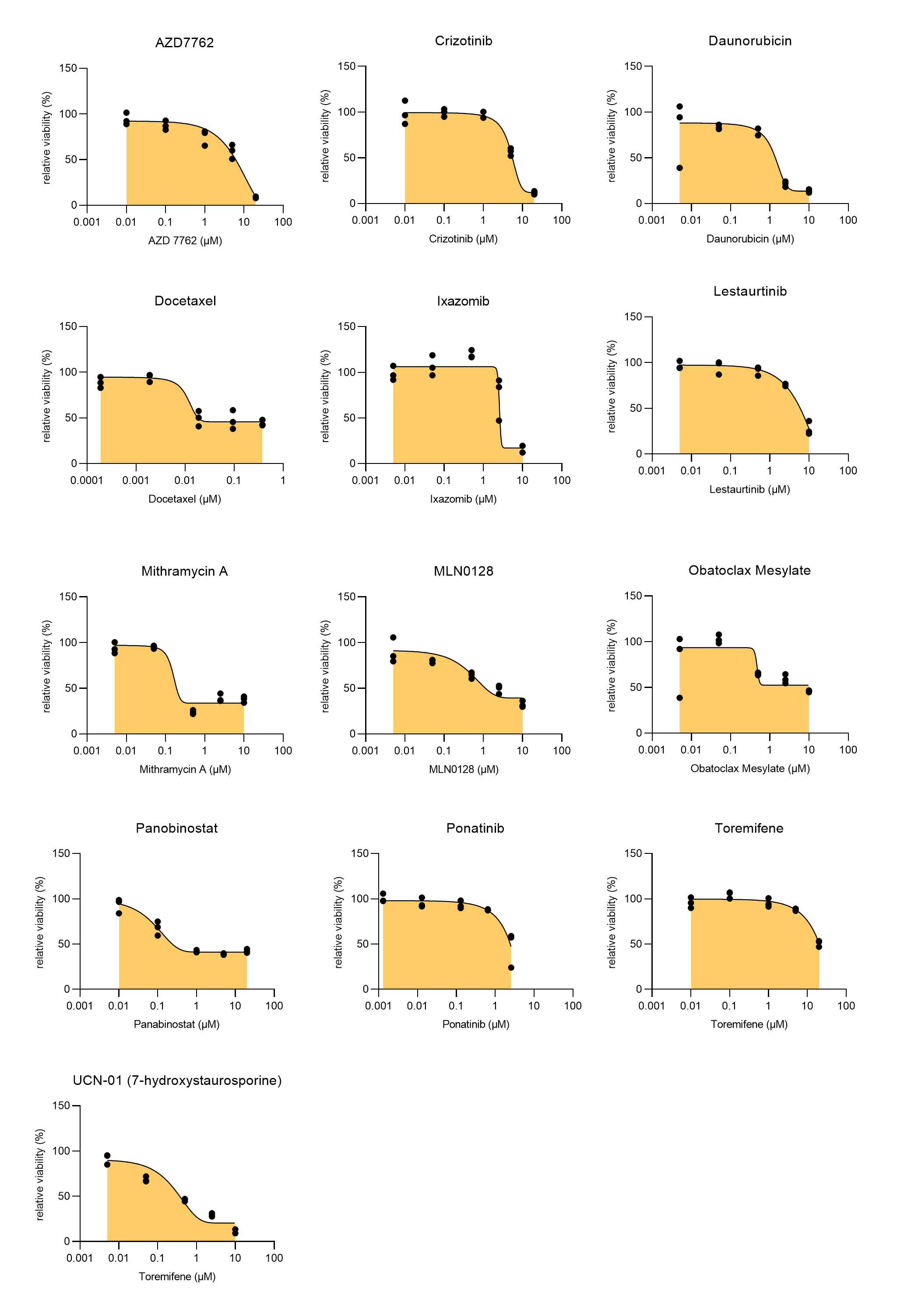

Supplement: Supplementary file 1 [file ijms-25-13315-s001.zip › Supplementary Figure S2..tif]
